# Associations Between Parental Employment and Children’s Screen Time: A Longitudinal Study of China Health and Nutrition Survey

**DOI:** 10.3389/ijph.2022.1605372

**Published:** 2023-01-10

**Authors:** Qian-Wen Xie, Xiangyan Luo, Roujia Chen, Xudong Zhou

**Affiliations:** ^1^ Department of Social Welfare and Risk Management, School of Public Affairs, Zhejiang University, Hangzhou, China; ^2^ Center of Social Welfare and Governance, Zhejiang University, Hangzhou, China; ^3^ Institute for Common Prosperity and Development, Zhejiang University, Hangzhou, China; ^4^ Future Regional Development Laboratory, Research Center for Common Prosperity, Innovation Center of Yangtze River Delta, Zhejiang University, Jiaxing, China; ^5^ The Institute of Social and Family Medicine, School of Medicine, Zhejiang University, Hangzhou, China; ^6^ The Second Affiliated Hospital, Zhejiang University School of Medicine, Hangzhou, China

**Keywords:** children, screen time, working hours, parental employment, informal employment, overwork

## Abstract

**Objectives:** Parents are often torn between their parenting roles in the family and working roles at the workplace. This study focused on the associations of parental employment with children’s screen time (ST) on weekdays, weekends, and during the entire week.

**Methods:** Unbalanced panel data including 2,977 children (aged 0–17 years) from five waves of the China Health and Nutrition Survey data from 2004 to 2015 were utilized. Two-way fixed effects models were fitted to examine the associations of parental employment status, working hours, and overwork with children’s ST.

**Results:** Compared to unemployment status, maternal formal employment positively predicted children’s ST on both weekdays and weekends, while maternal informal employment was associated with increased children’s ST on weekends. The more hours they worked, the more time their children spent using screens. Neither employment status nor the overwork of fathers was significant.

**Conclusion:** Parental employment, especially maternal employment, was linked with the ST of children. More childcare-friendly labor policies are needed to promote healthy lifestyles among the next generation.

## Introduction

Excessive screen time (ST) of children is associated with multiple negative physical and mental health consequences, such as obesity [1] and externalizing and internalizing symptoms [2]. As most of the screen use of children occurs within the home setting, parents have been widely recognized as “gatekeepers” in regulating children’s screen use [3]. General parenting styles and screen-specific parenting practices such as rule setting have been proven to be powerful predictors of children’s ST in previous studies [3–5]. In addition to the role of parents, it is important to note that people in the prime of life are also the major labor force in the market. More importantly, given the time and energy constraints, it is challenging for parents to simultaneously meet both work and family demands, especially their parenting duties [6]. Although it is acknowledged that parental employment benefits children by increasing family wealth, scholars have recently become concerned about its negative effects on childhood obesity [7] and weight-related lifestyles, including dietary intake, physical activity (PA), sedentary behavior, and sleep [8, 9]. The current study focused on children’s screen use and aimed at exploring the associations between parental employment, including both working hours and employment status, and the ST of their children.

Most likely because of the social norm that mothers take on the major caregiving responsibilities within families, the mainstream of currently available research in this field has concentrated on the influences of maternal employment, especially working hours, on their children’s ST. According to a recent systematic review, the majority of relevant studies reported positive associations between maternal working hours and children’s ST [8]. Non-etheless, mothers’ fewer working hours may not always be better. Their employment status may also matter, which is related to their financial stability and work schedule. For example, compared to children of mothers who were unemployed or worked full-time, children whose mothers took part-time jobs spent less time watching TV [10]. Another study found that children whose mothers worked under a non-standard work schedule (e.g., evening work) had longer ST than children of mothers who worked under a standard schedule (7 am–6 pm) [11].

Regardless of the number of working hours or employment status, limited attention has been given to the influence of paternal employment on children’s ST, let alone the impacts of the interplay of mothers and fathers [12]. In fact, increasing attention has been given to fathers involved in the discourse on parenting internationally [13]. Moreover, since children live in a complex and interactive family system, mother-child associations should be studied in the context of father-child associations and *vice versa* [14, 15]. It is worth noting that dual-earner families have become more predominant in both western and eastern societies as more women participate in the labor market than previously [10, 12, 16]. The whole family system may have to face an increasing challenge of work-family conflicts. From a family system perspective, therefore, it is of great importance to take the role of fathers and the interplay between parents into consideration when studying the impacts of parental employment on children’s ST.

Additionally, the potential differences in the association of parental employment with children’s ST between weekdays and weekends have been ignored, as previous studies commonly measured children’s ST with weekly total or seven-day average ST [8]. However, children’s screen use might demonstrate different patterns between weekdays and weekends because they would be impacted by kindergarten/school regimens during weekdays [3]. Additionally, children’s ST might be more influenced by parenting practices such as rule-setting or co-use on weekends since parents spend more time together with them [17]. More importantly, as the labor market continuously changes, new forms of employment, such as informal employment, have become available and popular among young generations due to flexibility in time arrangement [18, 19]. It is unclear whether parental employment will be differently associated with children’s weekday and weekend ST, especially when parents are informally employed and work outside normal working days.

In China, the female labor force participation rate is higher than the world average and regional average of East Asia [20]. Although there is still a traditional expectation of the male breadwinning role in Chinese culture, fathers have been expected to take on more caregiving duties at home as well under this context [13]. Meanwhile, the economic boom in recent decades has been accompanied by the problem of overwork in the Asia-Pacific region, including China [21]. To the best of our knowledge, no study has been conducted on the influences of parental employment on children’s ST in Chinese society. Therefore, with a nationally representative sample of Chinese children and parents, the current study aimed to examine: 1) the associations of paternal and maternal employment status with their children’s ST on weekdays, weekends, and in the entire week; 2) the associations of employed parents’ working hours and overwork with the weekday, weekend, and weekly total ST of their children; 3) whether these associations were moderated by children’s gender and age; and 4) whether the associations of mothers’ employment are moderated by fathers’ employment and *vice versa*.

## Methods

### Participants

We analyzed the data of the China Health and Nutrition Survey (CHNS) to achieve our research goals. The CHNS is a survey jointly conducted by the Demographic Research Center of the University of North Carolina and the Chinese Center for Disease Control and Prevention from 1989 to 2015. The CHNS covered 239 communities within 9 out of all 31 provinces that vary in demography, geography, economic development, and public resources in mainland China (Guangxi, Guizhou, Heilongjiang, Henan, Hubei, Hunan, Jiangsu, Liaoning, and Shandong). Based on a multistage random cluster sampling scheme, two cities and four counties were randomly selected from each province, and 10 villages and neighborhoods were selected from each county/city. Finally, 20 households in each village/neighborhood were selected for investigation. The investigation was approved by the Institutional Review Boards of the University of North Carolina at Chapel Hill and the National Institute of Nutrition and Food Safety, China Center for Disease Control and Prevention. Since questions about ST were not added to the survey until 2004, we utilized five waves (2004, 2006, 2009, 2011, and 2015) of data thereafter. All children under 18 years of age at the time of being surveyed and participating in at least two waves were sampled. Our final analyses were conducted on unbalanced panel data including 2,977 participants, with an average of 3.04 observations per subject (8,148 observations in total). Of these, 1,573 participants (38.61%) were tracked twice, 777 participants (28.61%) were tracked three times, 464 participants (22.78%) were tracked four times, and 163 participants (10%) were tracked five times.

### Measures

#### Screen Time

Self-reported or proxy-reported (for children under 6 years old) ST was collected in the CHNS. All participants were asked to report time spent on a typical weekday and weekend day in using different screen devices, including TV/videotapes, video games, computers, and mobile phones. Weekly total ST was the sum of two products: typical weekday ST × 5 (total weekday screen time) and typical weekend day ST × 2 (total weekend ST).

#### Parental Employment Status

Parents were asked to select their employment status from 6 options: 1) unemployed; 2) a permanent employee of an enterprise; 3) a contractor of an enterprise; 4) a self-employed worker; 5) a temporary worker; 6) a paid family worker; and 7) an unpaid family worker. A family worker refers to a person who worked for a company, enterprise, or business run by a family member or relative but did not have the right to make business decisions. Referring to the connotation of informal employment given by the International Conference of Labor Statisticians (ICLS) in 1993 and previous studies [22, 23], the current study categorized parental employment status into three types: unemployed (option 1), formal employment (options 2 and 3), and informal employment (options 4–7).

#### Parental Weekly Working Hours and Overwork

For employed parents, total working hours per week were computed by multiplying the number of working days per week by the number of working hours per day. According to the definition of overwork in previous American and Chinese studies [21, 24], working for more than 50 h per week was defined as overwork in the current study.

#### Covariates

Children- and family-level characteristics were used as covariates. Children’s factors included age, gender, ethnicity, residence, and PA. Self-reported or proxy-reported (for children under 6 years old) PA was collected. All participants were asked to report time spent on a typical weekday and weekend day in different physical activities, including walking, badminton, volleyball, football, basketball, tennis, and other sports. Weekly total PA was the sum of two products: typical weekday PA × 5 (total weekday physical activities time) and typical weekend day PA × 2 (total weekend physical activities time). Parental and family factors included parental education level (middle school and under/high school and technical school/college or above), inflation-adjusted family income *per capita*, the number of electronic devices owned by a household (including TV, computers, tablets, mobile phones, and VCD/DVD), and parental ST.

### Statistical Analyses

Descriptive analyses included sample counting and percentages of categorical variables, mean values, and standard deviations of continuous variables. Regression analyses were conducted in two steps. The first step was the regression of parental work on children’s ST with the entire sample (*n* = 7,623). The second step was the regression of parental working hours and overwork on children’s ST with the sample of employed parents (*n* = 5,061). In both steps, two-way fixed effects regression models were fitted. Since unmeasured personal characteristics and time-varying factors might affect children’s ST and parents’ work, the two-way fixed effects model could overcome potential bias caused by person-specific and time-specific differences. The regression equations were:

$$C{ST}_{it}=\alpha_{0}+\alpha_{1}{Employstatus}_{it}+\alpha_{2}X_{it}+a_{i}+\lambda_{t}+u_{it}$$

$$C{ST}_{it}=\beta_{0}+\beta_{1}{Workhours}_{it}+\beta_{2}X_{it}+a_{i}+\lambda_{t}+u_{it}$$

$$C{ST}_{it}=\gamma_{0}+\gamma_{1}{Overwork}_{it}+\gamma_{2}X_{it}+a_{i}+\lambda_{t}+u_{it}$$

where ${CST}_{it}$ denoted the ST (weekday, weekend, and weekly total) of child i in year t. Parental employment variable ${Employstatus}_{it}$ included parents’ employment status (unemployed, formal employment, and informal employment), ${Workhours}_{it}$ represented the working hours of parents, and ${Overwork}_{it}$ represented whether parents overworked. ‘0’ meant that the parents were not overworked, and ‘1’ meant that the parents were overworked. $X_{it}$ was a vector of control variables unaffected by prior treatment or outcomes. $a_{i}$ represented the individual fixed effect, $\lambda_{t}$ represented the time fixed effect, and $u_{it}$ represented a stochastic error term. The parameter $\alpha_{1}$ indicated the associations of parental employment status with children’s ST, parameter $\beta_{1}$ indicated the associations of parental working hours with children’s ST and parameter $\gamma_{1}$ indicated the associations of parental overwork with children’s ST. To further explore the effect mechanisms of parental employment on children’s ST, as long as the coefficients were significant in the base models, interaction terms were added to test the moderating effects of children’s age, gender, and the other parent’s employment.

Two sensitivity analyses were conducted to examine the robustness of our results, including the mean imputation to account for missing data and the examination of the non-linear associations. Parental age might affect not only the parental employment status and working hours but also their children’s ST. Nevertheless, parental age was not included in the main analyses as a control variable because it contained too much missing data. Therefore, in the first sensitivity analysis, we used the mean imputation method to fill in the missing values of parental age in each wave. We also explored the non-linearities between parental working hours and children’s ST.

All models were run in STATA 15 with the xtreg command, and *p* < .05 was the significance level. To make the effect sizes of different variables comparable, standardized regression coefficients were reported.

## Results

### Descriptive Statistics

The descriptive statistics of the key variables are shown in Table 1. The average age of the children in the full sample was 8.33 years (SD = 4.58). There was a gradual increase in ST among children, from 9.26 h per week (SD = 9.24) in 2004 to 14.78 h per week (SD = 14.43) in 2015. Children’s ST on weekends was longer than that on weekdays. Parental weekly total ST also presented an increasing trend from 2004 to 2015, and fathers had longer ST than mothers. In terms of the employment status of parents in the full sample, 14.46% of fathers and 30.06% of mothers were unemployed, 30.33% of fathers and 22.78% of mothers were formally employed, and 55.22% of fathers and 47.16% of mothers were informally employed. Among employed parents, on average, fathers worked 45.04 h per week (SD = 13.01), while mothers worked 40.79 h per week (SD = 15.60). Approximately 20% of employed parents (19.28% of fathers and 19.09% of mothers) overworked (worked more than 50 h per week).

**TABLE 1:** Characteristics of the study population (China Health and Nutrition Surveys, 2004–2015).

Wave	2004 (*n* = 1,411)	2006 (*n* = 1,698)	2009 (*n* = 1,599)	2011 (*n* = 1998)	2015 (*n* = 1,442)	Full sample (*n* = 8,148)
Variable	N (%)/Mean (SD)	N (%)/Mean (SD)	N (%)/Mean (SD)	N (%)/Mean (SD)	N (%)/Mean (SD)	N (%)/Mean (SD)
Children’s weekly total ST	9.26 (9.24)	11.25 (10.73)	12.46 (10.63)	11.81 (10.14)	14.78 (14.43)	11.90 (11.21)
Children’s weekday ST	5.75 (6.81)	6.78 (7.34)	7.38 (7.43)	7.03 (6.93)	9.16 (10.47)	7.20 (7.90)
Children’s weekend ST	3.65 (3.61)	4.41 (4.10)	5.05 (4.31)	4.85 (4.28)	5.42 (4.91)	4.69 (4.30)
Paternal employment status						
Unemployed	229 (16.23%)	215 (12.66%)	212 (13.26%)	213 (10.66%)	309 (21.43%)	1,178 (14.46%)
The formal employment	359 (25.44%)	421 (24.79%)	415 (25.95%)	744 (37.24%)	532 (36.89%)	2,471 (30.33%)
The informal employment	823 (58.33%)	1,062 (62.54%)	972 (60.79%)	1,041 (52.10%)	601 (41.68%)	4,499 (55.22%)
Maternal employment status						
Unemployed	456 (32.32%)	487 (28.68%)	454 (28.39%)	552 (27.63%)	500 (34.67%)	2,449 (30.06%)
The formal employment	239 (16.94%)	281 (16.55%)	307 (19.20%)	567 (28.38%)	462 (32.04%)	1856 (22.78%)
The informal employment	716 (50.74%)	930 (54.77%)	838 (52.41%)	879 (43.99%)	480 (33.29%)	3,843 (47.16%)
Paternal working hours	45.63 (13.56)	44.97 (13.12)	44.34 (13.97)	45.59 (12.11)	44.52 (12.36)	45.04 (13.01)
Maternal working hours	39.35 (15.81)	39.82 (16.88)	40.04 (17.52)	41.42 (14.86)	43.46 (11.38)	40.79 (15.60)
Paternal overwork						
No	927 (78.43%)	1,192 (80.38%)	1,098 (79.16%)	1,463 (81.96)	946 (83.50%)	5,626 (80.72%)
Yes	255 (21.57%)	291 (19.62%)	298 (20.84%)	322 (18.04%)	187 (16.50%)	1,344 (19.28%)
Maternal overwork						
No	778 (81.47%)	980 (80.92%)	892 (77.90%)	1,186 (82.02%)	775 (82.27%)	4,611 (80.91%)
Yes	177 (18.53%)	231 (19.08%)	253 (22.10%)	260 (17.98%)	167 (17.73%)	1,088 (19.09%)
Children’s age	7.59 (4.45)	8.69 (4.79)	8.30 (4.67)	7.41 (4.61)	9.94 (3.75)	8.33 (4.58)
Children’s gender						
Male	749 (53.08%)	905 (53.30%)	877 (54.85%)	1,051 (52.60%)	774 (53.68%)	4,356 (53.46%)
Female	662 (46.92%)	793 (46.70%)	722 (45.15%)	947 (47.40%)	668 (46.32%)	3,792 (46.54%)
Children’s ethnicity						
Han	1,171 (83.05%)	1,415 (83.33%)	1,338 (83.78%)	1732 (86.86%)	1,277 (88.74%)	6,933 (85.19%)
Minority	239 (16.95%)	283 (16.67%)	259 (16.22%)	262 (13.14%)	162 (11.26%)	1,205 (14.81%)
Residence						
Rural	1,082 (76.68%)	1,317 (77.56%)	1,235 (77.24%)	1,355 (67.82%)	955 (66.23%)	5,944 (72.95%)
Urban	329 (23.32%)	381 (22.44%)	364 (22.76%)	643 (32.18%)	487 (33.77%)	2,204 (27.05%)
Annual household income *per capita* (yuan)	4,237.76 (4,418.91)	4,799.65 (6,288.98)	7,815.08 (9,190.35)	10888.77 (12709.71)	17744.77 (26573.89)	9,063.32 (14639.95)
Father’s education						
Junior high and under	1,028 (72.86%)	1,132 (66.67%)	1,170 (73.17%)	1,246 (62.36%)	807 (55.96%)	5,383 (66.07%)
High school/Technical school	330 (23.39%)	462 (27.21%)	340 (21.26%)	490 (24.52%)	410 (28.43%)	2032 (24.94%)
College and above	53 (3.76%)	104 (6.12%)	89 (5.57%)	262 (13.11%)	225 (15.60%)	733 (9%)
Mother’s education						
Junior high and under	1,159 (82.14%)	1,331 (78.39%)	1,242 (77.67%)	1,365 (68.32%)	906 (62.83%)	6,003 (73.67%)
High school/Technical school	221 (15.66%)	294 (17.31%)	287 (17.95%)	369 (18.47%)	281 (19.49%)	1,452 (17.82%)
College and above	31 (2.20%)	73 (4.3%)	70 (4.38%)	264 (13.21%)	255 (17.68%)	693 (8.51%)
Number of electronic devices at home	1.67 (1.13)	1.84 (1.29)	2.16 (1.32)	2.43 (1.46)	4.31 (2.62)	2.45 (1.86)
Paternal weekly total ST	15.28 (13.51)	16.33 (12.17)	19.13 (15.37)	20.17 (14.27)	23.95 (19.77)	18.99 (15.37)
Paternal weekday ST	11.10 (10.87)	11.67 (9.63)	13.70 (11.93)	14.21 (10.79)	17.24 (15.70)	13.58 (12.00)
Paternal weekend ST	4.38 (3.45)	4.70 (3.37)	5.59 (4.20)	6.07 (4.38)	6.56 (5.39)	5.49 (4.28)
Maternal weekly total ST	14.20 (11.69)	14.40 (10.05)	17.88 (13.71)	18.13 (12.83)	21.42 (18.14)	17.21 (13.67)
Maternal weekday ST	10.14 (9.17)	10.09 (7.45)	12.67 (10.54)	12.88 (9.73)	15.47 (14.23)	12.24 (10.52)
Maternal weekend ST	4.17 (3.18)	4.30 (3.12)	5.28 (3.86)	5.38 (3.87)	5.76 (4.92)	4.99 (3.88)
Children’s weekly total PA	1.23 (3.67)	1.24 (3.43)	1.53 (3.98)	1.26 (3.42)	2.87 (5.70)	1.57 (4.07)
Children’s weekday PA	.84 (2.61)	.87 (2.49)	1.08 (2.88)	.89 (2.62)	2.18 (4.43)	1.12 (3.05)
Children’s weekend PA	.41 (1.28)	.40 (1.19)	.43 (1.22)	.42 (1.23)	.70 (1.64)	.46 (1.31)

ST, screen time; PA physical activity.

### The Associations Between Parental Employment Status and Children’s ST

The associations between parental employment status and their children’s ST are shown in Table 2. Compared to children with unemployed mothers, children whose mothers were formally employed had longer ST on weekdays (*α* = 1.10, 95% CI [.32, 1.89], *p* < .01), weekends (*α* = .47, 95% CI [.06, .88], *p* < .05) and during the entire week (*α* = 1.53, 95% CI [.43, 2.63], *p* < .01), and children whose mothers had informal work had longer weekend ST (*α* = .35, 95% CI [.05, .64], *p* < .05). No significant correlation was found between paternal employment status and children’s ST. Furthermore, children’s age and gender moderated the associations between maternal employment status and children’s ST. Compared to 0- to 5-year-old children, mothers’ formal employment status was less associated with older children’s weekday ST (6- to 11-year-olds: *α* = −2.93, 95% CI [−4.03, −1.83], *p* < .001; 12- to 17-year-olds: *α* = −4.56, 95% CI [−6.01, −3.11], *p* < .001), weekend ST (12- to 17-year-olds: *α* = −1.09, 95% CI [−1.84, −.33], *p* < .01) and weekly total ST (6- to 11-year-olds: *α* = −3.16, 95% CI [−4.71, −1.61], *p* < .001; 12- to 17-year-olds: *α* = −5.41, 95% CI [−7.45, −3.37], *p* < .001). Mothers’ informal employment status had a larger impact on the weekend ST of 6- to 11-year-olds (*α* = .60, 95% CI [.19, 1.00], *p* < .01), but a smaller impact on the weekend ST of 12- to 17-year-olds (*α* = −.64, 95% CI [−1.21, −.07], *p* < .05) than the ST of the 0- to 5-year-olds. A positive interaction term between mothers’ formal employment status and the male gender of children (*α* = 2.40, 95% CI [.52, 4.29], *p* < .05) indicated that boys were more strongly associated with maternal employment status and the association mainly appeared on weekends (*α* = .77, 95% CI [.07, 1.47], *p* < .05). No significant interaction effect was observed between paternal and maternal employment status.

**TABLE 2:** Regression analysis of parental employment status on children’s screen time (China Health and Nutrition Surveys, 2004–2015).

	Dependent variable: Children’s ST								
	Weekday ST			Weekend ST			Weekly total ST		
	Estimate	95% CI		Estimate	95% CI		Estimate	95% CI	
		Lower	Upper		Lower	Upper		Lower	Upper
Base models									
Paternal employment status (ref.: unemployed)									
Formal employment	−.71	−1.53	.12	.12	−.31	.55	−.80	−1.96	.37
Informal employment	−.05	−.77	.67	.29	−.08	.67	.10	-.91	1.11
Maternal employment status (ref.: unemployed)									
Formal employment	1.10**	.32	1.89	.47*	.06	.88	1.53**	.43	2.63
Informal employment	.40	−.17	.97	.35*	.05	.64	.78	-.01	1.58
*F* Statistic	27.66***(18,4632)			43.39(18,4632)			36.49***(18,4632)		
*R* ^*2*^	.10			.14			.12		
	**Models with children’s age as moderator (ref.: children aged 0–5)**								
Mother formally employed × Children aged 6–11	−2.93***	−4.03	−1.83	−.57	−1.15	.01	−3.16***	−4.71	−1.61
Mother formally employed × Children aged 12–17	−4.56***	−6.01	−3.11	−1.09**	−1.84	-.33	−5.41***	−7.45	−3.37
*F Statistic*	28.71***(20,4630)			39.50***(20,4630)			34.42***(20,4630)		
*R* ^*2*^	.11			.15			.13		
Mother informally employed × Children aged 6–11				.60**	.19	1.00			
Mother informally employed × Children aged 12–17				−.64*	−1.21	−.07			
*F Statistic*				41.26***(20,4630)					
*R* ^*2*^				.15					
	**Models with children’s gender as moderator (ref.: girls)**								
Mother formally employed × Boys	1.19	−.15	2.53	.77*	.07	1.47	2.40*	.52	4.29
*F Statistic*	26.38***(19,4631)			41.39***(19,4631)			34.93***(19,4631)		
*R* ^*2*^	.10			.15			.13		
Mother informally employed × Boys				−.17	−.68	.34			
*F Statistic*				41.13***(19,4631)					
*R* ^*2*^				.14					
	**Models with the other parent’s employment as moderator**									
Mother formally employed × Father formally employed	.01	−1.20	1.23	−.40	−1.03	.24	−.56	−2.27	1.15
*F Statistic*	26.20***(19,4631)			41.19***(19,4631)			34.58***(19,4631)		
*R* ^*2*^	.10			.14			.12		
Mother informally employed × Father informally employed				.04	−.48	.56			
*F Statistic*				41.10***(19,4631)					
*R* ^*2*^				.14					

Significance: *p < .05; **p < .01; ***p < .001.

CI, confidence interval; ST, screen time.

Adjustment for children’s age, gender, ethnicity, residence, physical activity, parental education and ST, logarithmic family income *per capita* and the number of electronic devices owned by a household. Significant results are highlighted in bold.

### The Associations Between Employed Parents’ Working Hours and Children’s ST

Table 3 presents the association of the working hours of employed parents with children’s ST. The mothers’ working hours increased the time that their children spent using screens on weekdays (*β* = .04, 95% CI [.02, .06], *p* < .001), weekends (*β* = .01, 95% CI [.00, .03], *p* < .01), and during the entire week (*β* = .06, 95% CI [.03, .09], *p* < .001). On the contrary, children whose fathers worked longer had shorter ST on weekday (*β* = −.03, 95% CI [−.05, −.001], *p* < .05). Moderation analyses showed that 0- to 5-year-old children were more inclined to be affected by maternal working hours than older peers with regard to weekly total, weekday, and weekend ST. On the contrary, the weekday ST of older children had a stronger negative association with their father’s working hours compared to 0- to 5-year-old children. The moderating effects of the child’s gender and the other parent’s working hours were insignificant.

**TABLE 3:** Regression analysis of parental working hours on children’s screen time (China Health and Nutrition Surveys, 2004–2015).

	Dependent variable: Children’s ST								
	Weekday ST			Weekend ST			Weekly total ST		
	Estimate	95% CI		Estimate	95% CI		Estimate	95% CI	
		Lower	Upper		Lower	Upper		Lower	Upper
Base models									
Paternal working hours	−.03*	−.05	−.001	−.005	−.02	.01	−.03	−.07	.00
Maternal working hours	.04***	.02	.06	.01**	.00	.03	.06***	.03	.09
*F Statistic*	13.50***(16,2524)			22.20***(16,2524)			17.58***(16,2524)		
*R* ^*2*^	.08			.12			.10		
	**Models with children’s age as the moderator (ref.: children aged 0–5)**								
Maternal working hours × Children aged 6–11	−.05***	−.07	−.02	.00	−.02	.01	−.04**	−.07	−.01
Maternal working hours × Children aged 12–17	−.09***	−.12	−.06	−.03**	−.04	−.01	−.12***	−.16	−.07
*F Statistic*	13.58***(18,2522)			21.40***(18,2522)			17.32***(18,2522)		
*R* ^*2*^	.09			.13			.11		
Paternal working hours × Children aged 6–11	−.04***	−.06	−.02						
Paternal working hours × Children aged 12–17	−.08***	−.11	−.04						
*F Statistic*	13.13***(18,2522)								
*R* ^*2*^	.09								
	**Models with children’s gender as the moderator (ref.: girls)**								
Maternal working hours × Boys	.01	−.03	.05	.01	-.01	.03	.02	-.04	.08
*F Statistic*	12.72***(17,2523)			20.97***(17,2523)			16.57***(17,2523)		
*R* ^*2*^	.08			.12			.10		
Paternal working hours× Boys	−.01	−.06	.04						
*F Statistic*	12.72***(17,2523)								
*R* ^*2*^	.08								
	**Models with the other parent’s working hours as the moderator**								
Maternal working hours × Paternal working hours	−.000	−.001	.00	−.000	−.001	.00	−.000	-.001	.00
*F Statistic*	12.71***(17,2523)			20.96***(16,2524)			16.56***(16,2524)		
*R* ^*2*^	.08			.12			.10		

Significance: *p < .05; **p < .01; ***p < .001.

CI, confidence interval; ST, screen time.

Adjustment for children’s age, gender, ethnicity, residence, physical activity, parental education and ST, logarithmic family income *per capita* and the number of electronic devices owned by a household. Significant results are highlighted in bold.

### The Associations Between Employed Parents’ Overwork and Children’s ST

As shown in Table 4, children with overworked mothers had longer weekly total ST (*γ* = 2.51, 95% CI [1.34, 3.69], *p* < .001), as well as children’s ST during weekdays (*γ* = 1.60, 95% CI [.74, 2,47], *p* < .001) and weekends (*γ* = .85, 95% CI [.42, 1.28], *p* < .001). Children’s age moderated the associations of maternal overwork with children’s weekday and weekly total ST but failed to moderate the association with weekend ST. The moderating effects of gender were not significant. The analysis of the interaction between paternal and maternal overwork identified a significant moderating effect of paternal overwork on the association between maternal overwork and children’s ST. Specifically, children whose parents both overworked spent less time on screens during weekdays and the entire week than their peers with only one or none of the parents overworked (weekday ST: interaction γ = −1.64, 95% CI [−3.00, −.29], *p* < .05; weekly total ST: interaction γ = −2.28, 95% CI [−4.13, −.44], *p* < .05).

**TABLE 4:** Regression analysis of parental overwork on children’s screen time (China Health and Nutrition Surveys, 2004–2015).

	Dependent variable: Children’s ST								
	Weekday ST			Weekend ST			Weekly total ST		
	Estimate	95% CI		Estimate	95% CI		Estimate	95% CI	
		Lower	Upper		Lower	Upper		Lower	Upper
Base models									
Paternal overwork	−.44	−1.31	.44	−.12	−.56	.32	−.66	−1.86	.53
Maternal overwork	1.60***	.74	2.47	.85***	.42	1.28	2.51***	1.34	3.69
*F Statistic*	13.39***(16,2524)			22.78***(16,2524)			17.83***(16,2524)		
*R* ^*2*^	.08			.13			.10		
	**Models with children’s age as the moderator (ref.: children aged 0–5)**								
Maternal overwork × Children aged 6–11	−1.69*	−3.33	−.06	.28	−.54	1.09	−1.21	−3.43	1.02
Maternal overwork × Children aged 12–17	−3.40**	−5.50	−1.30	−.95	−2.00	.10	−4.35**	−7.22	−1.49
*F Statistic*	12.49***(18,2522)			20.76***(18,2522)			16.44***(18,2522)		
*R* ^*2*^	.08			.13			.11		
	**Models with children’s gender as the moderator (ref.: girls)**									
Maternal overwork × Boys	−.29	−1.97	1.39	−.13	−.97	.70	−.44	−2.73	1.84
*F Statistic*	12.60***(17,2523)			21.44***(17,2523)			16.78***(17,2523)		
*R* ^*2*^	.08			.13			.10		
	**Models with the other parent’s overwork as the moderator**								
Maternal overwork × Paternal overwork	−1.64*	−3.00	−.29	−.61	−1.28	.07	−2.28*	−4.13	-.44
*F Statistic*	13.70***(16,2524)			22.98***(16,2524)			18.15***(16,2524)		
*R* ^*2*^	.08			.13			.10		

Significance: *p < .05; **p < .01; ***p < .001.

CI, confidence interval; ST, screen time.

Adjustment for children’s age, gender, ethnicity, residence, physical activity, parental education and ST, logarithmic family income *per capita* and the number of electronic devices owned by a household. Significant results are highlighted in bold.

### Results of Sensitivity Analyses

As shown in Table 5, the regression results based on the imputed dataset did not suggest an apparent difference from the main analyses. The results in Table 6 implied that no evidence was found for non-linear associations between parental working hours and children’s ST.

**TABLE 5:** Sensitivity analysis (I) – Regression analyses based on the sample with mean-imputed parental age (China Health and Nutrition Surveys, 2004–2015).

	Dependent variable: Children’s ST								
	Weekday ST			Weekend ST			Weekly total ST		
	Estimate	95% CI		Estimate	95% CI		Estimate	95% CI	
		Lower	Upper		Lower	Upper		Lower	Upper
	**Regression analysis of parental employment status on children’s screen time (n = 7,623)**								
Paternal employment status (ref.: unemployed)									
Formal employment	−.75	−1.58	.07	.10	−.33	.54	−.88	−2.04	.29
Informal employment	−.09	−.81	.62	.28	−.10	.65	.03	−.98	1.04
Maternal employment status (ref.: unemployed)									
Formal employment	1.10**	.31	1.88	.47*	.06	.88	1.53**	.43	2.63
Informal employment	.41	-.16	.98	.35*	.06	.65	.80*	.01	1.60
*F Statistic*	25.37***(20,4630)			39.33*** (20,4630)			33.50***(20,4630)		
*R* ^*2*^	.10			.15			.13		
	**Regression analysis of parental working hours on children’s screen time (*n* = 5,061)**								
Paternal working hours	−.03*	−.05	−.001	−.005	−.02	.01	−.03	−.07	.00
Maternal working hours	.04***	.02	.06	.01*	.00	.03	.05***	.02	.08
*F Statistic*	12.26***(18,2522)			19.82***(18,2522)			15.90***(18,2522)		
*R* ^*2*^	.08			.12			.10		
	**Regression analysis of parental overwork on children’s screen time (n = 5,061)**								
Paternal overwork	−.39	−1.27	.49	−.12	−.56	.32	−.61	−1.81	.58
Maternal overwork	1.62***	.75	2.48	.86***	.43	1.29	2.55***	1.37	3.73
*F Statistic*	12.17***(18,2522)			20.37***(18,2522)			16.16***(18,2522)		
*R* ^*2*^	.08			.13			.10		

Significance: *p < .05; **p < .01; ***p < .001.

CI, confidence interval; ST, screen time.

Adjustment for children’s age, gender, ethnicity, residence, physical activity, parental education and ST, logarithmic family income *per capita* and the number of electronic devices owned by a household. Significant results are highlighted in bold.

**TABLE 6:** Sensitivity analysis (II) – Test for non-liner associations between parental working hours and children’s screen time (China Health and Nutrition Surveys, 2004–2015).

	Dependent variable: Children’s ST								
	Weekday ST			Weekend ST			Weekly total ST		
	Estimate	95% CI		Estimate	95% CI		Estimate	95% CI	
		Lower	Upper		Lower	Upper		Lower	Upper
Paternal working hours	−.03*	**−**.05	**−**.001	**−**.004	**−**.02	.01	**−**.03	**−**.07	.00
Maternal working hours	.04***	.02	.06	.01**	.00	.03	.06***	.03	.09
*F Statistic*	13.50***(16,2524)			22.20***(16,2524)			17.58***(16,2524)		
*R* ^*2*^	.08			.12			.10		
Paternal working hours	**−**.02	**−**.10	.05						
Maternal working hours	.00	**−**.06	.07	**−**.02	**−**.05	.02	**−**.01	**−**.10	.07
Paternal working hours^2^	**−**.000	**−**.001	.00						
Maternal working hours^2^	.00	**−**.000	.00	.00	**−**.000	.00	.00	**−**.000	.00
*F Statistic*	12.06***(18,2521)			21.08***(17,2522)			16.66***(17,2522)		
*R* ^*2*^	.08			.12			.10		

Significance: *p < .05; **p < .01; ***p < .001.

CI, confidence interval; ST, screen time.

Adjustment for children’s age, gender, ethnicity, residence, physical activity, parental education and ST, logarithmic family income *per capita* and the number of electronic devices owned by a household. Significant results are highlighted in bold.

## Discussion

Prolonged ST is highly prevalent among children worldwide, and parents are the most important “gatekeepers” in regulating their children’s screen use [3]. However, parents are often torn between the roles of being parents in the family and employees in the job market [6]. Based on the five-wave survey data of a large sample of Chinese children and parents (N = 2,977), the current study explored the association between parental employment, including employment status, working hours, and overwork, and their children’s ST, with specific attention to the ST in the entire week, on weekdays, and on weekends.

Maternal employment has been the primary focus in this field [8, 10]. The current study identified the strong linkages between mothers’ employment and children’s ST across models of employment status, working hours, and overwork. First, this study added to the literature on maternal employment’s impacts on children’s ST by specifying the different effects of formal and informal employment status. Compared to children with unemployed mothers, children whose mothers were formally employed had longer ST on both weekdays and weekends, while maternal informal employment status was only associated with children’s weekend ST. Although women have been able to enter the workplace since the Industrial Revolution, they are not liberated from family labor so far [25]. Working mothers usually participate in the first shift as employees in their workplaces to bring earnings into the household while take the second shift (e.g., household duties and caregiving) as the main force at home [9, 26]. Informally employed mothers are more prone to work outside regular workdays and might have more weekend work than formally employed ones [27]. Their employment was likely to decrease their time with children during weekends, only influencing children’s weekend ST. Formally employed mothers were usually busy with their work schedules during weekdays and might also have to hold the unpaid work responsibilities (e.g., housework) after work. They would let children do some screen activities instead of engaging in interactions with their children on both weekdays and weekends, due to time pressure [12].

The current study also found that, for employed mothers, the more hours they worked, the more time their children spent using screens on both weekdays and weekends. Compared to children with mothers who did not overwork, those with mothers who worked more than 50 h per week had longer ST during both weekdays and weekends. The ST of younger children (0–5 years old) was more inclined to be associated with maternal employment status, working hours, and overwork than older children, as they might be more dependent on maternal supervision [12]. According to the “scarcity hypothesis,” it is difficult for people to satisfy all demands from different roles [28]. Employed mothers, especially those who were formally employed or had younger children, may face great challenges in balancing their working duties and child-rearing responsibilities, such as supervising children’s screen use and physical activities. Many studies have shown the relationship between maternal employment and childhood obesity [7]. Results of the current study provided a possible explanation for the pathway through which maternal employment impacted their children’s weight from the perspective of children’s ST.

Comparatively, we found that neither employment status nor the overwork of fathers was significant in any regression models. Moreover, more paternal working hours were associated with less children’s weekday ST. In fact, paternal working hours have been commonly treated as a control variable in previous studies on the impact of maternal employment on children’s ST, while a significant paternal influence has rarely been reported [8]. A very recent study began to highlight the impacts of paternal employment on children’s ST and found that children whose fathers were employed had shorter ST than children whose fathers were unemployed in Japan [12]. The authors argued that great psychosocial stress on unemployed fathers might prevent them from performing caregiving duties due to the traditional expectations of the “male breadwinner” in Japanese society. Like in other East Asian countries, the traditional expectations of the “male breadwinner” and female housekeeper are still dominant in China [13, 29]. It is not surprising that maternal employment exerted a greater influence than paternal employment on children’s screen use in China, as women have long been assumed to be the major caregivers at home. As the main breadwinner in the family, longer working time of employed fathers might bring more financial resources into the household which could allow them to enroll their children in more extracurricular physical activities, leading to less children’s ST. As a result, families and society might place a greater emphasis on the important role of mothers rather than fathers in child-rearing responsibilities, which might further lead to greater gender inequality in labor market.

Since China has a fairly high female labor participation rate, dual-earner couples are becoming more prevalent [20]. A previous study hypothesizes that there is an interplay of parental employment when affecting children’s ST [12]. With a small sample of children (N = 866), this cross-sectional study failed to identify a significant interaction effect in the regression analysis. With a larger longitudinal sample, the current study examined parent-child dyadic relationships in the context of the other parent. Although both the interaction between maternal and paternal employment status and the interaction between maternal and paternal working hours were insignificant, it was intriguing to find that children whose parents both overworked had shorter weekday and weekly total ST than their peers who only had one or neither parent who overworked. A possible explanation could be that complementary childcare, particularly from grandparents, might be sought when both parents are busy with their work [30]. Grandparenting is especially common in contemporary dual-earner families in China, with approximately 60% of elders having once taken care of their grandchildren [31]. The presence of grandparents in the households might be beneficial to preventing children from sedentariness [32]. More notably, this finding revealed no simple linear relationship between parental working hours and children’s ST. Relevant factors within the family system, such as complementary childcare, should be further explored in future studies.

Empirical analyses using two-way fixed effect models based on the nationally representative panel data enabled this study to make causal inferences. Nevertheless, some limitations must be noted. First, self- or proxy-reported ST was used as the main outcome, so the results were likely to suffer from information bias. Future research should be conducted with other data that are very detailed at capturing young people’s daily routines, such as the 24-Hour Diary and Ecological Momentary Assessment (EMA) [33–35]. Also, the current study only focused on the duration of children’s screen use, ignoring the contents they watched. Future studies are needed to explore the relationship between parental employment and the contents that their children watch, as children’s screen use behaviors are much more complicated than ST. Second, the impact of parents’ work schedules on children’s ST could not be identified in the current study as our dataset only provided the information regarding the weekly total working hours of parents without distinguishing weekdays and weekends. Future investigations might need to measure parents’ working hours more flexibly by taking their work schedules into consideration. Third, single-parent families were excluded from the current study. Maternal and paternal employment were simultaneously placed in the models as we would like to examine the interactive effects of parental employment on children’s ST in a family system. The impact of parental employment on children’s ST in single-parent families should be explored in future studies. Fourth, the current study did not examine the potential effect mechanisms. Childcare was the most likely effect channel, but the data quality of relevant variables was relatively poor in the CHNS, which made the examination infeasible. Fifth, children’s ST might be affected by factors besides parental employment, as effect sizes were relatively small in our models. More comprehensive research on children’s ST is needed in the future. Last, as this study adopted data covering 2004 to 2015, it might not reflect the *status quo* of the current job market in China. Also, this data failed to involve the boom of mobile devices in recent years. Future studies using more updated data are recommended.

### Conclusion

Based on the longitudinal data from the CHNS, the current study explored the associations of parental employment with their children’s ST. The findings of this study highlighted the detrimental impacts of work-family conflict commonly faced by Chinese mothers, especially those who were formally employed or had younger children, on their children’s health-related lifestyles. Chinese fathers should be encouraged to take more child-rearing responsibilities at home. Moreover, making the next-generation healthier is not merely a family issue. National work-family policies should work toward a childcare-friendly path, which would rescue parents, especially mothers, from the dilemma of balancing their work and family. Strengthening childcare support in the community and wider society is essentially important for supporting working families’ needs and improving children’s health in the long run.

## Abbreviations

CHNS, China Health and Nutrition Survey; PA, physical activity; ST, screen time.
